# Chemotherapy as Treatment for Acute Myeloid Leukemia (AML)-Induced Facial Nerve Palsy

**DOI:** 10.7759/cureus.23710

**Published:** 2022-03-31

**Authors:** Michael Fana, Brook Centofanti, Philip Kuriakose

**Affiliations:** 1 Neurology, Henry Ford Health System, Detroit, USA; 2 Hematology and Medical Oncology, Henry Ford Health System, Detroit, USA

**Keywords:** neuroradiology, neurooncology, bell's palsy, mastoid surgery, acute myeloid leukemia (aml)

## Abstract

Acute myeloid leukemia (AML) is a disorder of the myeloid cell line that can manifest infrequently as a granulocytic sarcoma with infiltration into bone and soft tissue. Consequently, cranial nerve neuropathy due to AML infiltration can result in variable neurological deficits, including facial nerve palsy. Here, we present the case of a patient presenting with unilateral facial nerve palsy with evidence of AML in cerebrospinal fluid (CSF) cytology and bilateral opacification of the mastoid air cells suggestive of AML infiltration into the mastoid process. Patient demonstrated improvement of facial palsy after administration of intrathecal chemotherapy without need for surgical intervention. We further examine known cases reported to date on the use of chemotherapy and surgical intervention in management of facial nerve palsy as a consequence of AML infiltration of the mastoid bone. Notably, there appears to be a correlation between mastoid bone infiltration seen on imaging and facial nerve palsy in patients with known history of AML that may be treated without need for surgical intervention or biopsy.

## Introduction

Acute myeloid leukemia (AML) is a disorder of the myeloid cell line that results in bone marrow failure with the presence of increased blasts, anemia and thrombocytopenia. Granulocytic sarcoma is an infrequent manifestation of AML, in which immature myeloid cells infiltrate bone and soft tissue. These extra-medullary infiltrates are associated with poorer survival rates in adults with AML [1]. As a result of the rarity of this manifestation of AML in less than 1% of patients with extra-medullary AML, the prognostic factors are undetermined. In one large retrospective study, median survival of patients with extra-medullary granulocytic sarcoma as a manifestation of AML was 10.7 months [2]. Such infiltrative manifestations can cause compression of cranial nerves resulting in variable neurologic deficits, including facial nerve palsy which has rarely been described in association with other cranial nerve deficits such as otalgia, vertigo and abducens palsy [3]. Here, we present the case of a patient presenting with unilateral Bell's palsy with evidence of AML in cerebrospinal fluid (CSF) cytology and bilateral opacification of the mastoid air cells.

## Case presentation

Our patient is a middle-aged female with a history of acute myeloid leukemia of complex karyotype (inv(16), KIT+) diagnosed two years prior. Since diagnosis, she had completed chemotherapy with cytarabine and idarubicin (7+3) and azacytidine before receiving allogenic stem cell transplant. Patient was in remission but relapsed several months later and was re-started on azacytidine and venetoclax with remission. Her medical course was then complicated further by another relapse associated with new cerebral myeloid sarcoma formation and leptomeningeal spinal metastasis resulting in functional quadriplegia. She underwent successful external bream radiation of the frontal lobe sarcoma with evidence of a complete response and started intrathecal chemotherapy with alternating methotrexate and cytarabine twice a week until CSF was clear of blasts and was then switched to once a week, followed by once a month, regimen.

Our patient presented to us two weeks after having missed her last planned chemotherapy session with signs of acute onset right-sided facial drooping. She scored an initial IV on House-Brackmann facial paralysis scale indicating moderately-severe dysfunction apparent by the asymmetric appearance of the mouth, nasolabial fold, eyebrows, and eyes with trace visible movement on examination and incomplete closure of the eyelid at rest. She denied any hearing impairment, vision changes, or facial sensory deficits. All other cranial nerve, head, ear, nose, and throat exam findings were unremarkable. Laboratory assessment found no evidence of leukocytosis with an unremarkable peripheral blood smear. Initial CT head and neck, CT perfusion, and CT angiography were unremarkable for acute ischemic events, masses, or vascular malformations. CSF analysis demonstrated leukocytosis with 92% blasts on cytology. Workup for viral, bacterial, and fungal CSF infections were negative.

**Figure 1:**
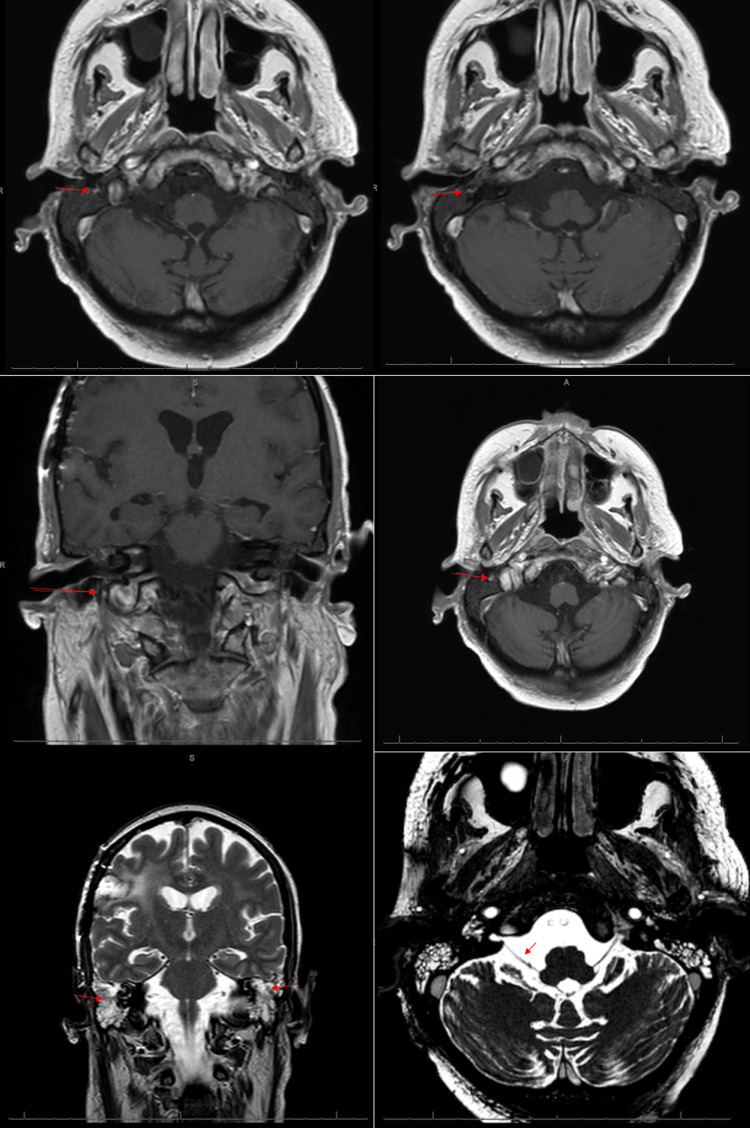
MRI brain with contrast Top Row: T1-weighted axial cross-sections demonstrating a focal area of high signal intensity seen adjacent to the right facial nerve. Middle Row: T1-weighted coronal and axial cross-sections demonstrating minimal enhancement of the descending intra-mastoid segment distally of the right facial nerve just proximal to the stylomastoid foramen. Bottom Row: T2-weighted coronal cross-section demonstrating right post-surgical encephalomalacia and opacification of the bilateral mastoid air cells (left). Axial cross-section demonstrating patency of cranial nerve VII exiting the pons (right).

An MRI with vestibular protocol for cranial nerve assessment was acquired and demonstrated complete opacification of the mastoid middle ear cavities bilaterally. There was normal fluid signal intensity seen within the inner ear structures, normal partitioning of the cochlea, and no enlargement of the endolymphatic duct or sac. There was no enlargement of the facial nerve root. Notably, there was minimal enhancement of the descending intra-mastoid segment distally of the right facial nerve proximal to the stylo-mastoid foramen when compared to the left shown in Figure 1.

Our patient was started on a four-day course of IV dexamethasone along with weekly alternating intrathecal chemotherapy with methotrexate and cytarabine. She received one round of methotrexate and demonstrated improvement three days later in facial palsy to Class II House-Brackmann classification notable by near symmetry of facial expression at rest with only subtle weakness of the mouth, nasolabial fold, and eyebrows and eye movement and near-complete closure of the eyelid at rest. However, five days, later she decompensated secondary to aggressive disease relapse complications and passed.

## Discussion

The facial nerve arises at the level of the pons within the brainstem and protrudes caudally at the cerebellopontine angle to course through the internal acoustic meatus and enter the facial canal within the petrous portion of the temporal bone. It then exits through the stylo-mastoid foramen dividing into multiple branches, including those implicated in motor control of the facial muscles which divide at the posterior aspect of the parotid gland [4]. Though our patient’s MRI did not demonstrate any obvious sign of facial nerve inflammation or compressive neuropathy from mass effect (e.g., a myeloid sarcoma), there was notable enhancement of the descending intra-mastoid segment of the nerve adjacent to the stylo-mastoid foramen suggesting an inflammatory process. Moreover, given the overt opacification of the bilateral mastoid processes, our suspicion is that of AML relapse as indicated on lumbar puncture (LP) results with infiltration of the mastoid air cells which may be implicated in the neuropathic cranial nerve inflammation appreciated within the mastoid bone.

To date and to our knowledge, there have been few cases of AML infiltration into the mastoid process implicated as the cause of facial nerve palsy. Through a thorough literature review of 178 results in PubMed for “Leukemia” and “Facial nerve palsy”, we were able to identify 11 case reports on AML presenting with facial nerve palsy, the results of which are listed in Table 1. Of the 11 case reports, five cases reported the utility of surgical resection and biopsy of the mastoid bone with findings of a myeloid sarcoma in some instances. While two of these cases saw no resolution of their facial nerve palsy following surgery, the other three responded only after chemotherapy [5-9]. On the other hand, five cases reported resolution of the facial palsy after starting chemotherapy without need for surgical intervention [10-14].

**Table 1:** Summary of Reported Symptoms, Outcomes, Operative Approaches, and Medical Management of Facial Nerve Palsy Associated with AML in Current Literature. N/A: not applicable

Article	Age(s)	Presenting Symptom(s)	Outcome	Facial Palsy Resolution After Chemotherapy	Presence of Mastoiditis	Surgical Intervention(s)	Cranial Imaging Findings	Chemotherapy Regimen
Ranta et al.[3]	Toddlers	Unilateral facial palsy (6 patients); Others: Abducens palsy, headache, otalgia, vertigo, cauda equine syndrome	Outcome was alive in 4, death in 5	Not reported	Yes (7 patients)	None	CT and MRI head: bilateral mastoid air cell opacification in 5 of 6 patients with facial palsy	Not reported
Leite da Silveira et al.[5]	Female Adult	Unilateral facial palsy; Bilateral otalgia with sensorineural hearing loss	Partial resolution of facial palsy after antrostomy. Patient deceased 13 days after start of chemotherapy from neutropenia and sepsis	Partial	Yes - Bilateral	Antrostomy	CT head: bilateral opacification with increased soft tissue density and bilateral dehiscence of the facial canal in the tympanic portion of the facial nerve	Systemic daunorubicin and cytarabine
Levy et al.[6]	Female Child	Unilateral facial palsy; Mastoiditis	Resolved facial palsy	Yes	Yes	Mastoidectomy	X-ray showed poor pneumatization of both mastoid bones	Cytosine arabinoside and daunorubicin
Nishioka et al.[7]	Male Adult	Unilateral facial palsy; Otalgia	No resolution of facial palsy Mild improvement in otalgia	No	Yes - Unilateral	Mastoidectomy	CT head: opacified mastoid air cells and occlusion of external auditory canal	Neocarzinostatin, BHAC, and prednisolone
Todd et al.[8]	Male Teenager	Unilateral facial palsy; Otalgia	Resolved facial palsy	Yes	Yes - Unilateral	Mastoidectomy	N/A	Systemic cytarabine, thioguanine, daunorubicin. Intrathecal methotrexate, hydrocortisone, cytosine arabinoside
Zappia et al.[9]	Female Child	Unilateral facial palsy; Otalgia	Resolved facial palsy	Yes	Yes - Unilateral	Mastoidectomy	CT head: opacified left mastoid air cells	Not disclosed
Eser et al.[10]	Female Teenager	Unilateral facial palsy; Paraplegia of lower extremities	Resolved facial palsy	Yes	Yes - Bilateral	None	MRI head: bilateral maxillary sinus involvement of tumor and mastoiditis	Adriamycin and cytarabine
Kubota et al.[11]	Male Infant	Unilateral facial palsy; Otalgia	Resolved facial palsy	Yes	No	None	MRI and CT head: No intracranial abnormalities	Systemic etoposide, cytarabine and mitoxantrone
Lee et al.[12]	Female Adult	Unilateral facial palsy; Bilateral optic neuritis	Resolution of facial palsy and visual acuity	Yes	No	None	CT head: No intracranial abnormalities	Intrathecal methotrexate and cytarabine
Sandal et al.[13]	Female Adult	Unilateral facial palsy	Resolved facial palsy	Yes	No	None	N/A	Dasatinib dexamethasone and vincristine
Takhenchangbam et al.[14]	Male Infant	Unilateral facial palsy; Unilateral eye proptosis	Partial remission of proptosis Resolution of facial palsy	Yes	No	None	CT head and MRI of orbits: polypoidal soft tissue mass in the left maxillary antrum showing pressure erosions of smooth thinning of the medial, posterolateral walls and boxy orbital floor at places extending into the inferior extraconal space of the left orbit with mild proptosis.	Systemic daunorubicin and cytarabine
Current Report	Female Adult	Unilateral facial palsy	Mild improvement of facial palsy. Patient deceased 5 days after start of chemotherapy from aggressive AML relapse	Partial	Yes - Bilateral	None	MRI head: opacification of the mastoid middle ear cavities bilaterally and minimal enhancement of the descending intra-mastoid segment distally of the right facial nerve proximal to the stylo-mastoid foramen	Intrathecal methotrexate

In our patient, we suspect the etiology of the opacification of the mastoid air cells to be that of leukemic infiltration as the patient did not demonstrate clinical evidence of infectious mastoiditis and therefore did not require biopsy of the bone for assessment. Moreover, in our patient and many other patients with leukemia, risk of surgical procedures are considerably elevated due to higher incidences of infections and bleeds which outweigh the risk-to-benefit ratio of the procedure. Our clinical decision was against systemic chemotherapy for our patient at the time of presentation since she did not have clinical evidence of bone marrow or systemic relapse and she presented with an overall poor health condition and functional status. Although intrathecal chemotherapy was started, we did not anticipate resolution of facial droop, particularly if it was due to an inflammatory or compressive neuropathy of the facial nerve within the mastoid process as the chemotherapy would poorly penetrate the mastoid air cells. However, as in other cases reported to date, our patient did demonstrate improvement in her facial palsy to House-Brackmann class II after starting intrathecal methotrexate. In particular, we noted improvement in facial symmetry with only mild weakness in facial expression as well as ability to close her eye while at rest and during sleep. One other report to date has demonstrated similar results with methotrexate therapy in conjunction with cytarabine and indicate resolution of Bell's palsy and visual symptoms in the patient [12]. However, the standard therapeutic protocol for AML treatment remains unchanged per patient regardless of symptoms. As highlighted in Table 1, the most common therapy for AML involves use of cytarabine and an anthracycline, such as daunorubicin, which is well documented in literature as the classic "7+3" treatment regimen for AML [15]. Nonetheless, the specific choice of chemotherapy will be patient specific and may, for instance, incorporate medications such as methotrexate in patients without resistance in their genetic profile. Therefore, resolution of facial nerve palsy symptoms is likely dependent on the treatment of AML as opposed to a specific chemotherapy regimen. In our patient, we anticipated further improvement and possible recovery with weekly intrathecal methotrexate. However, our patient unfortunately rapidly decompensated from complications of AML relapse.

## Conclusions

While facial nerve palsy is a rare presentation of AML, we have demonstrated here in our patient case and in others reported in literature that the presence of mastoid air cell opacification may be an indication of possible disease manifestation or relapse in patients in remission when presenting with facial nerve palsy. Moreover, resolution of the palsy is possible with intrathecal chemotherapy alone without need for surgical intervention or biopsy of the mastoid process. While the chemotherapy regimen may be patient specific, further studies will be required as to the efficacy and duration of the chemotherapy needed for complete facial nerve palsy resolution. We describe here evidence of improvement in facial motor weakness after intrathecal methotrexate in a patient presenting with Bell's palsy secondary to AML.
